# Protein disulfide isomerase uses thrombin–antithrombin complex as a template to bind its target protein and alter the blood coagulation rates

**DOI:** 10.1042/BSR20231540

**Published:** 2024-05-15

**Authors:** Abdul Burhan Khan, Urfi Siddiqui, Sana Fatima, Ahmed Abdur Rehman, Mohamad Aman Jairajpuri

**Affiliations:** Department of Biosciences, Jamia Millia Islamia, Jamia Nagar, New Delhi 110025, India

**Keywords:** Anticoagulant, Antithrombin, Protein Disulfide Isomerase, thrombin, Thrombosis

## Abstract

During inflammation and situations of cellular stress protein disulfide isomerase (PDI) is released in the blood plasma from the platelet and endothelial cells to influence thrombosis. The addition of exogenous PDI makes the environment pro-thrombotic by inducing disulfide bond formation in specific plasma protein targets like vitronectin, factor V, and factor XI. However, the mechanistic details of PDI interaction with its target remain largely unknown. A decrease in the coagulation time was detected in activated partial thromboplastin time (APTT), prothrombin time (PT), and thrombin time (TT) on addition of the purified recombinant PDI (175 nM). The coagulation time can be controlled using an activator (quercetin penta sulfate, QPS) or an inhibitor (quercetin 3-rutinoside, Q3R) of PDI activity. Likewise, the PDI variants that increase the PDI activity (H399R) decrease, and the variant with low activity (C53A) increases the blood coagulation time. An SDS-PAGE and Western blot analysis showed that the PDI does not form a stable complex with either thrombin or antithrombin (ATIII) but it uses the ATIII–thrombin complex as a template to bind and maintain its activity. A complete inhibition of thrombin activity on the formation of ATIII–thrombin–PDI complex, and the complex-bound PDI-catalyzed disulfide bond formation of the target proteins may control the pro- and anti-thrombotic role of PDI.

## Introduction

Human protein disulfide isomerase (PDI) is predominantly an endoplasmic reticulum (ER) based thiol isomerase that catalyzes the disulfide bond formation in nascent protein chains, it also has a chaperone-like function [1,2]. During venular inflammation, hypoxia, or excessive free radical generation the platelet and endothelial cell granules have been shown to release PDI in blood plasma [3–5]. Several potential substrates in plasma have been identified using PDI-trap variants that have been shown to influence thrombosis [6]. Thus, the PDI-based disulfide bond formation of pro- and anticoagulant factors was proposed as one of the mechanisms of its prothrombotic role, however, the molecular details of such complexes are largely unexplored. Additionally, there is conflicting evidence about the role of PDI in thrombosis. While a research group demonstrated that PDI accumulation was associated with thrombus formation using an *in vivo* mice model [7], the PDI inhibitors completely inhibited arterial thrombus formation and platelet aggregation. Another *in vitro* study showed that it has anticoagulant nature [8]. Contradictory reports also exist regarding the role of PDI in cellular response to stress, while some claim its up-regulation to be protective [9,10], others have shown it to be detrimental to cell survival [11].

There are several proteins that have been identified to be part of coagulation and hemostasis that have been characterized as PDI substrates. Platelet-dependent human thrombin (FIIa) generation was first shown to be dependent on the PDI-mediated activation of platelet factor V (FV) to release it from multimurin-1, which when complexed with FV inhibits thrombus formation [12,13]. Also, an increased rate of conversion of factor XI (FXI) to FXIa following its reduction by PDI was shown to enhance the cleavage of its physiological substrates, Factor IX (FIX) [14]. PDI was shown to catalyse the formation of a ternary complex with antithrombin–thrombin (ATIII–FIIa) [15,16]. The interaction of PDI with thrombospodin1 (TSP1) has been shown to increase the adhesion of endothelial cells to TSP1 via the αvβ3 integrin receptor [17,18]. PDI’s procoagulant profile signifies that silencing or inhibition of PDI decreased coagulation activity, whereas the addition of exogenous PDI increased its coagulation activity [19]. The mechanism points to an intricate regulation of the components that control coagulation and anticoagulation pathways. It seems like antithrombin (ATIII) modulation by PDI may directly affect the coagulation rates and rate of thrombus formation. Therefore, understanding the regulation of coagulation and thrombus mediated through PDI and ATIII is critical to assess their mechanism and predict the potential of PDI-based control on thrombosis. A preliminary study has revealed that ATIII–FIIa is one of the targets of PDI-based modulation, as it may shift the prothrombotic balance under stress to maintain hemostasis. We show a possible mechanism where PDI activity is maintained on binding to the ATIII–thrombin complex which it may use as a template to bind PDI plasma targets.

## Materials and methods

### Generation of recombinant human PDI

The gene for human PDI in the pUC57 vector was purchased from GenScript Biotech Corporation, New Jersey, U.S. Later, it was sub-cloned into the pET-28b (+) (Novagen, U.S.A.) within the Nhe I and Hind III restriction sites, a 6X His-tagged was also placed at the N-terminal. To get the optimum expression in a bacterial expression system, the codon was optimized. The resulting gene was transformed in *Escherichia coli DH5α* competent cells to amplify the copy number of the plasmid. Colonies were picked, followed by incubation in the primary culture, and then the plasmid was isolated by using a mini-prep plasmid isolation kit (Qiagen, Hilden, Germany). Plasmid was run on 0.8% agarose gel before and after digestion with Nhe I and Hind III restriction enzymes (Supplementary Figure S1A and 1B), which was further confirmed by Sanger's sequencing performed by Macrogen, Seoul, South Korea.

### Preparation of N- and C-terminal catalytic site variants of PDI

Catalytic site cysteines at position Cys 53 were substituted to Ala and His 399 to Arg on the wild type (wt) PDI, with an additional six-His tag at the N-terminus in pET-28b (+) vector (Novagen, U.S.A.) by site-directed mutagenesis using Phusion High-fidelity DNA polymerase F530S (Thermo fisher scientific). The PDI gene was used as a template, complementary mutagenic primers were employed to amplify the variants (Supplementary Table S2, inset). PCR reaction condition included an initial denaturation for 300 s of 1 cycle at 94°C, followed by a second denaturation at 94°C for 40 s of the 16–18 cycles, and annealing at gradient temperature for 40 s, of 16–18 cycles and 480 s extensions at 72°C followed by final extension of the last cycle prolonged to 600 s at 72°C. The parental plasmid was removed from the PCR product using the Dpn I enzyme, and the PCR products were then transformed into DH5α competent cells. DNA sequencing provided the proof of the mutation.

### Expression and purification of the recombinant proteins

The *Escherichia coli* BL21 (DE3) cells (NEB) expressing the wtPDI and its variant of interest, were treated in primary culture with 50 μg/ml kanamycin sulfate (HiMedia) for approximately 10–12 h at 37°C at moderate shaking of approximately 130–140 rpm. From overnight grown primary media, 0.1% was transferred in the 2000 ml of Luria Bertani broth, Miller (HiMedia) containing 50 μg/ml kanamycin sulfate and incubated at 37°C for 3–4 h. At a cell density (OD_600_) of 0.6 overexpression was induced using 0.5 mM IPTG, incubated for 12–14 h at 20°C with vigorous shaking (180–190 rpm). Centrifugation was used to collect overnight-grown cells, which were then reconstituted in 50 ml of Lysis buffer (30 mM Tris-HCl pH 8.0, 300 mM NaCl, 0.5% Triton X-100, 1 mM PMSF, 0.1 μg/ml lysozyme, 5% Glycerol) followed by 20 rounds of sonication on the ice at 20 s pulses and 20 s rest (Sonics Vibracell VCX750, Newtown, Ct, U.S.A.). Cellular lysates were clarified by centrifugation at 12,000 × ***g*** for 40 min at 4°C and passed over a His-Select Ni-affinity resin (GE Healthcare, U.S.A.) with a 3 ml bed volume. A wash buffer (30 mM Tris-HCl pH 8.0, 500 mM NaCl, 20 mM Imidazole, 0.5% Triton X-100, 5% glycerol) was used to wash the affinity resin, followed by another wash using 3 column volumes of wash buffer containing (30 mM Tris-HCl pH 8.0, 300 mM NaCl, 20 mM Imidazole, 0.5% Triton X-100, 5% glycerol). The fractions were eluted using 50 mM-1.0 M gradients of imidazole wash buffer. Purified monomeric protein was buffer exchanged (137 mM NaCl, 2.7 mM KCl, 10 mM Na_2_HPO_4_, 1.8 mM KH_2_PO_4_, pH 7.4) using Amicon Ultra-15 centrifugal 30 kDa concentrator (Millipore, Merck), after examining the purity on the 10% non-denaturing gel.

### Purification of ATIII from fresh plasma

The human plasma was used to purify the ATIII by utilizing a 5 ml HiTrap Heparin HP affinity column (Cytiva). Human plasma was diluted with 1X PNE buffer (4.4 mM NaH_2_PO_4_.H_2_O, 15 mM Na_2_HPO_4_, 100 mM, 200 mM EDTA) in a 1:2 ratio and then loaded onto the Hi-Trap affinity chromatography column [20]. Before loading the plasma, the column was equilibrated with 1× PNE and then eluted with different NaCl concentration gradients (0–3.0 M). Eluted fractions were run on 10% SDS-PAGE to check the purity of the ATIII (Supplementary Figure S3). Human plasma was obtained from Rotary Bank, New Delhi, and ethical clearance for the same was obtained from the Institute ethical committee, JMI.

### Assay for enzyme-dependent disulfide reduction of insulin

The assay examines the kinetics of protein aggregation in which PDI facilitates the reduction of insulin in the presence of dithiothreitol (DTT). The aggregates were seen spectrophotometrically at 650 nm since PDI significantly reduces the β-chain of insulin. The assay was performed as described by [19], with a modest adjustment to the reaction conditions and concentrations on a 96-well plate. The assay buffer consisted of 100 mM potassium phosphate and 0.2 mM EDTA, pH 7.0. Insulin with a final concentration of 0.16 and 175 nM of PDI in 100 µl of total volume was added. 1 mM of DTT was used from the stock of 20 mM to initiate the reaction. The sample was incubated at 25°C for 30 min before monitoring at 650 nm on SpectroMax M2 (Molecular Devices, Sunnyvale, CA, U.S.A.). All the reactions contained, 175 nM wtPDI and its variants, when incubated the concentration of the ATIII was 350 nM, the FIIa concentration was 18 nM and that of the vitronectin was 175 nM.

### Antithrombin inhibition assay

To assess the specificity of wtPDI and its mutant on ATIII, FIIa, and AIII–FIIa complex in the presence or the absence of vitronectin (Uniport accession ID-P04004), an ATIII-based inhibition of FIIa was performed [21]. The concentration of wtPDI, ATIII, FIIa, and vitronectin was 175 nM, 350 nM, 18 nM, and 175 nM, respectively. All the reactions were performed for 30 min and the sample was withdrawn after every 3 min. FIIa was already placed in all the microtiter well plates and wtPDI under reduced conditions together with 1 mM GSH was incubated for 0-30 min. The inhibition reaction was at 25°C for 30 min and then stopped by the addition of 150 μM of the chromogenic substrate S-2238. The chromogenic substrate served a dual purpose of halting the inhibition reaction by competition and providing a means of quantitating the residual activity due to uninhibited FIIa.

### Fluorescence spectroscopy

On a Jasco FP-6300 spectrofluorometer, a fluorescence measuring experiment for wtPDI and its cysteine, and histidine variants was performed to assess the structural differences between wtPDI and the variants. Samples were excited at 280 nM and fluorescence spectra of the 3 μM protein sample were recorded from 300 to 450 nm with an excitation and emission slit width of 5 nm. All reactions were performed at 25°C in 20 mM sodium phosphate buffer (pH 7.4). The buffer spectra were subtracted to correct all fluorescence spectra.

### Circular dichroism study

The far UV-CD spectra of wtPDI and the variants were studied using a Peltier temperature controller-equipped Chirascan Plus CD spectropolarimeter from Applied Photophysics. A 25°C temperature, a scan speed of 100 nm/min, and a response time of 1 s were used for CD analysis. For this experiment, the concentration was kept at 2 mg/ml. Samples were prepared in 20 mM sodium phosphate buffer, pH 7.4. Scanning of spectra was done between 260 and 190 nm. Each spectrum indicated the strongest signal, which was calculated as the average of 10 separate scans after baseline adjustment.

### *In vitro* clotting assays

Citrated vials containing a 3.8% sodium citrate solution were used to collect human blood. Blood was centrifuged for 20 min at 2400 × ***g*** to extract platelet-poor plasma. Activated partial thromboplastin time (APTT), prothrombin time (PT), and thromboplastin time (TT) clotting assays were performed by utilizing commercially available kits and in accordance with the manufacturer's instructions. All the experiments were performed by the previously described protocol [22,23]. Each test run was repeated three times with 175 nM of wtPDI and its variants were used during the clotting time experiments, where the protein was added exogenously. The protein was incubated with 100 µM of Q3R and QPS prior to its addition to the coagulation assay. Appropriate buffers in the absence of test samples were taken and baseline correction for buffer contributions was done.

### Assessment of the PDI, thrombin, antithrombin, and vitronectin complexes

Interaction analysis of wtPDI and its variants with FIIa, ATIII, and vitronectin was performed with slight changes in the concentrations of the samples [15]. Approximately 250 nM of vitronectin, 375 nM of ATIII, 250 nM of FIIa, 1 mM GSH, and heparin (2 units/ml) were incubated in the presence of 250 nM wtPDI or its variants at 25°C. First, the ATIII–FIIa complex was formed in 30 min of incubation with heparin. In ATIII-FIIa interaction with the wtPDI and its variants, the mixture was incubated for 15 min. In the ATIII–FIIa–Vitronectin–PDI ternary complex formation, the vitronectin and wtPDI were mixed with the preformed ATIII–FIIa complex and incubated for another 15 min at 25°C. All the samples were prepared, and experiments were performed in non-reducing conditions by using a complex buffer (10 mM HEPES, 137 mM NaCl, 4 mM KCl, and 15 mM glucose [pH 7.4, containing 2 mM CaCl_2_]). The reaction was stopped by the addition of a gel sample buffer containing 2% SDS. The samples for determining the PDI, FIIa, or ATIII activities were also prepared similarly. The samples were also analyzed using 7.5% homogeneous SDS-PAGE with a 4.5% stacking gel. The running samples were stained by using nimble juice (GeneDireX, inc.) fast and sensitive fluorescent dye for visualization and quantitation of the protein samples.

### Western blot analysis

Complexes of the wtPDI and its variants and their interactions with the FIIa, ATIII, and vitronectin were analyzed by Western blot. PDI and ATIII were probed simultaneously using 1:1000 dilution of mouse anti-P4HB PDI antibody [RL90] (Abcam, U.S.A.) and 3 µg/ml of mouse anti-human ATIII antibody, and then visualized by 1:1500 dilution of goat anti-mouse IgG, (H+L), peroxidase-conjugated. Pierce ECL Western blotting substrate was used in the 1:100 dilution range.

### Statistics

GraphPad Prism 5 was used for the statistical analysis of the data. The data were presented as the mean ± SEM, one-way ANOVA, and Tukey’s test for several groups for parametric comparison.

## Results

### PDI modulation of the blood coagulation time

**Figure 1:**
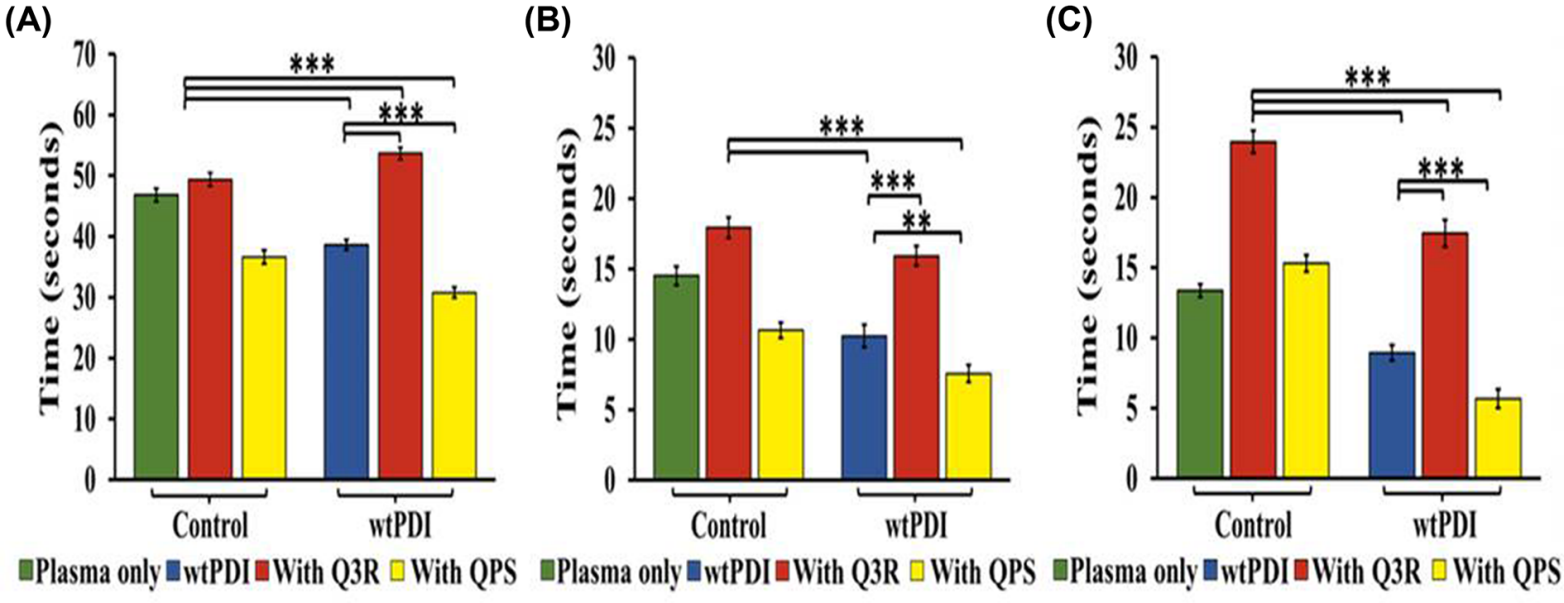
Effect of Q3R and QPS on coagulation time In the control set, from A to C, the green bar represents the negative control having platelet-poor plasma (PPP) only, while the red and yellow bar represents the PPP with Q3R and QPS respectively. Within the wtPDI set, the blue bar represents the wtPDI in platelet-poor plasma. The red bar denotes the presence of Q3R with PPP and wtPDI, and the yellow bar indicates the incubation of QPS with PPP and wtPDI. (**A**) Activated partial thromboplastin time (APTT), (**B**) Prothrombin time (PT), and **(C)** thrombin time (TT) show the delay in clotting time in the presence of Q3R (100 µM) when incubated with wtPDI (175 nM) which is already known inhibitor of PDI; however, incubation of wtPDI with QPS (100 µM) fasten the clotting time. *P*-values of <0.05 were considered as significant with *P<0.05, **P<0.01 and ***P<0.001, ANOVA. Data are representative of at least five independent experiments (*n* = 5 in each group).

Control of PDI activity in blood plasma can assist in regulating the clotting time. Consequently, the clotting time was measured while utilizing an activator (QPS) and an inhibitor (Q3R) of PDI. The summarized results can be observed in Figure 1. The addition of the PDI to blood plasma results in a partial decrease in clotting time in both the activated prothrombin time (APTT) and prothrombin time (PT) representing the intrinsic and extrinsic blood coagulation pathways, respectively. These clotting times are further decreased in the presence of PDI activator QPS and increased in the presence of its inhibitor rutin (Q3R) (Figure 1A,B). However, the effects were more pronounced in the common pathway represented by the thrombin time (TT) (Figure 1C). In the thrombin time assay (TT), clotting time decreased by 33.13%, corresponding to reductions of approximately 17.44% in APTT and 29.44% in PT assay. Likewise, QPS exhibited a significant decrease in clotting time in the TT assay by 62.96%, with reductions of approximately 20.27% in APTT and 34.16% in the PT assay (Supplementary Table S3). These results point to a direct effect of the exogenously added PDI on the mechanism associated with the thrombin-dependent coagulation cascade.

### Site-directed mutagenesis and purification of the PDI variants

**Figure 2:**
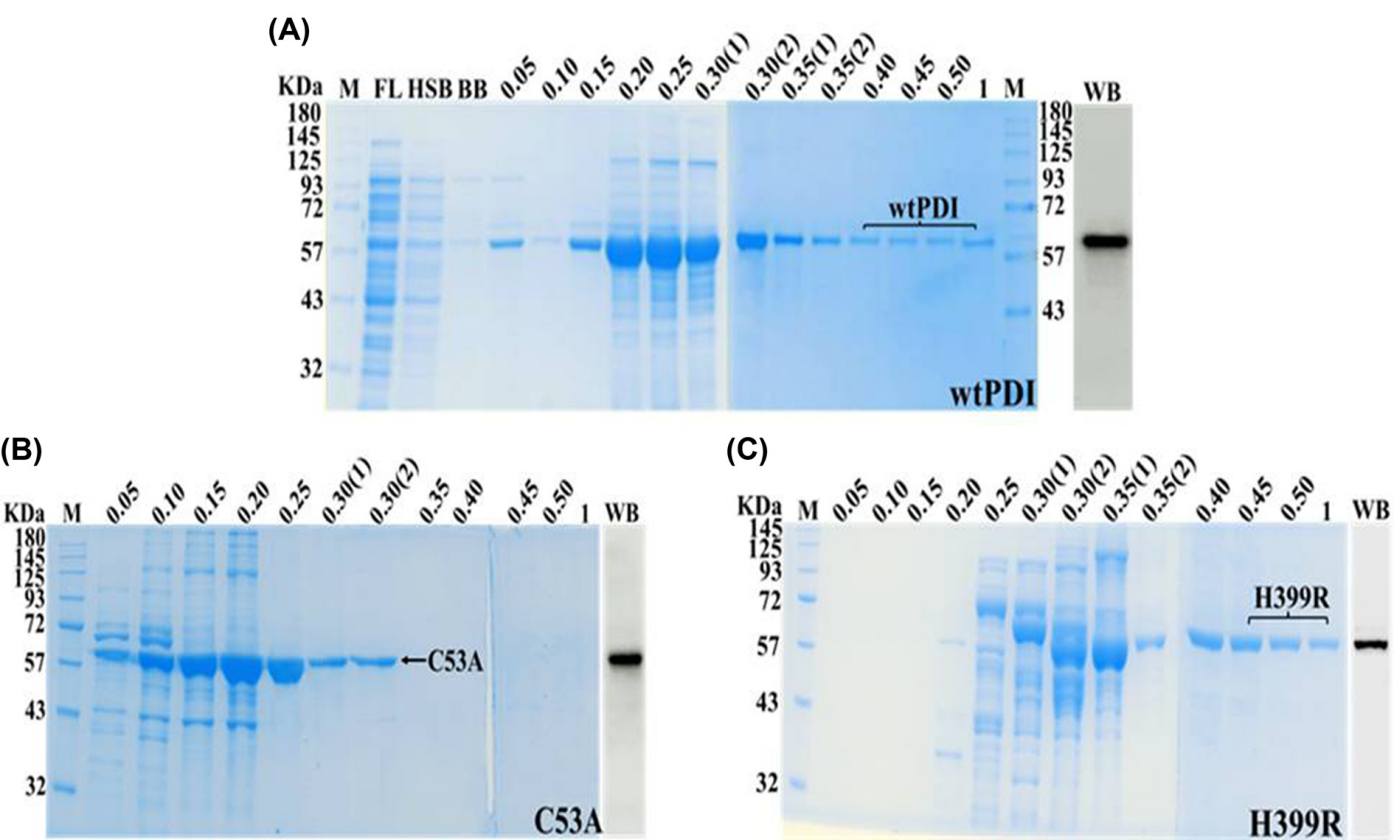
Purification of wtPDI and its variants wtPDI and its variants were purified using affinity chromatography, (**A–C**) representing the purified protein fractions on SDS-PAGE and Western blot analysis. All the fractions were eluted in 0.05 M to 1 M imidazole. **Lane M** is an 11–180 kDa protein marker, **FL** (used in A) represents flow through, and **Lane HSB** and **BB**, of (A) stand for high salt buffer fraction and binding buffer fraction. **Lane WB** represents the Western blot, which was performed by using the Abcam’s anti-PDI antibody.

We designed N and C terminal PDI variants intending to get variants with low and high activities and possibly use them as traps for binding PDI substrates. PDI variants Cys53Ala and His399Arg were made by site-directed mutagenesis using conditions and the reagents shown in Supplementary Tables S1 and 2 in a PCR reaction based on one tube PCR analysis and the confirmation of the variant (Supplementary Figure S2). WtPDI and its mutated constructs C53A and H399R, were purified and the purity was determined by 10% SDS PAGE (Figure 2). SDS PAGE gels of wtPDI (Figure 2A), C53A (Figure 2B), and H399R (Figure 2C) were used to pool and concentrate the monomeric fractions and confirmed with the Western blot analysis (Figure 2, inset). The pooled fractions were kept at −80°C for further usage. ATIII was also purified using the Hi-trap heparin column using standard purification protocol (data not shown).

### Activity and structural analysis of the PDI variants

**Figure 3:**
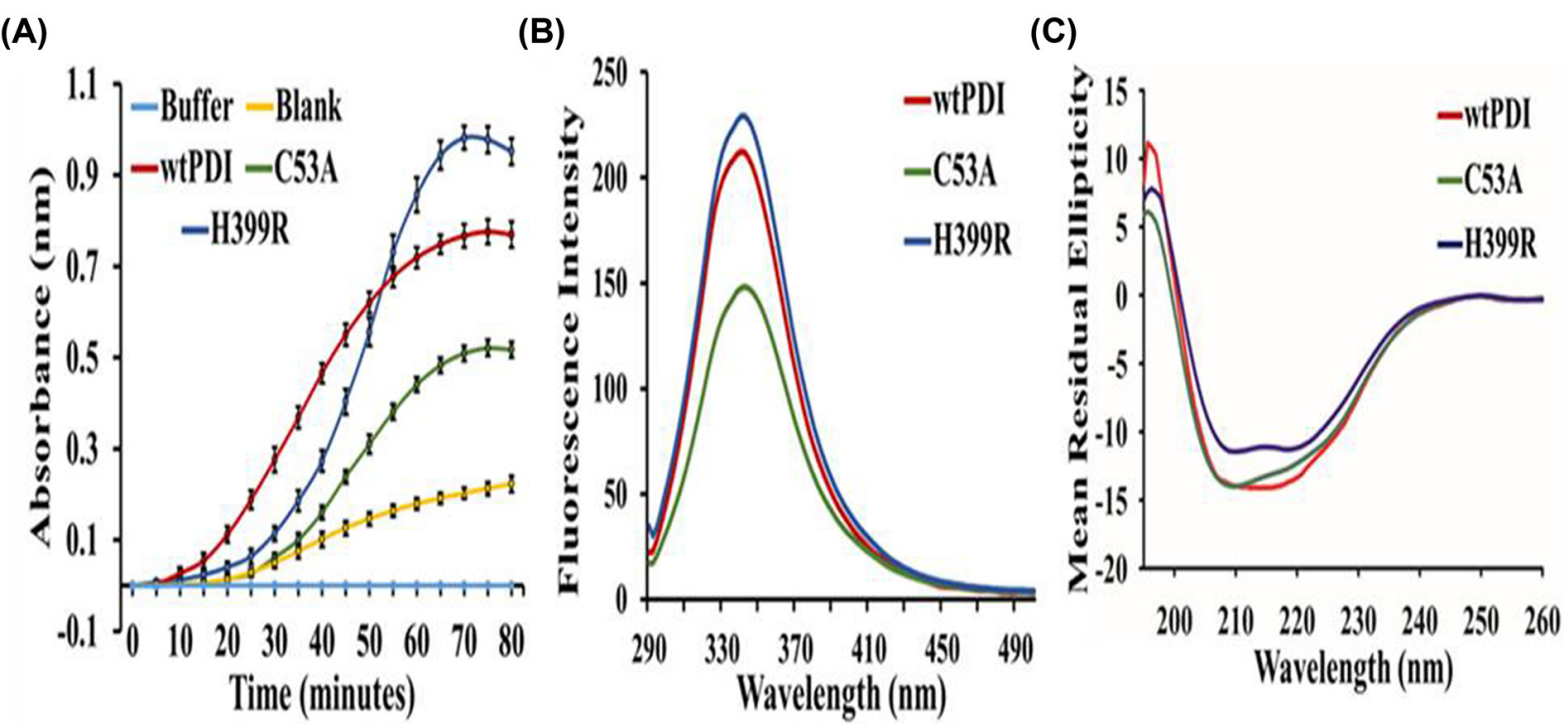
Effect of conserved residue mutations on PDI insulin reductase activity, and its secondary and tertiary structures (**A**) Insulin reductase activity of wtPDI and its variants, Cys53Ala and His399Arg. Panel (**B**) represents the change in fluorescence intensity of wtPDI and its variants, at the maximum fluorescence emission spectra at 340 nm. Panel (**C**) represents the circular dichroism spectra of wtPDI and its variants, the change in the α-helical content was monitored at 208 and 222 nm. All the plots are an average of at least three independent experiments.

PDI activity measurements are done in partially reducing conditions using an insulin turbidity assay, in which PDI facilitates the reduction of insulin in the presence of the reducing agent dithiothreitol (DTT). We observed that the H399R PDI variant showed enhanced activity, while C53A showed a decrease in PDI activity (Figure 3A). Fluorometric analysis of the wtPDI and its variant showed that the emission intensity of H399R was comparable; however, C53A showed a decrease in the emission intensity as compared with the wtPDI control (Figure 3B). The secondary structure of the variants of PDI were assessed using circular dichroism studies and the results showed that C53A has a comparable profile as compared with wtPDI; however, H399R showed a decrease in the α-helical content (Figure 3C). The data indicate that H399R showed an increase, while a C53A PDI variant showed a decrease in PDI activity, and the secondary structure of H399R was marginally compromised.

### Influence of PDI variant on the blood coagulation rates

**Figure 4:**
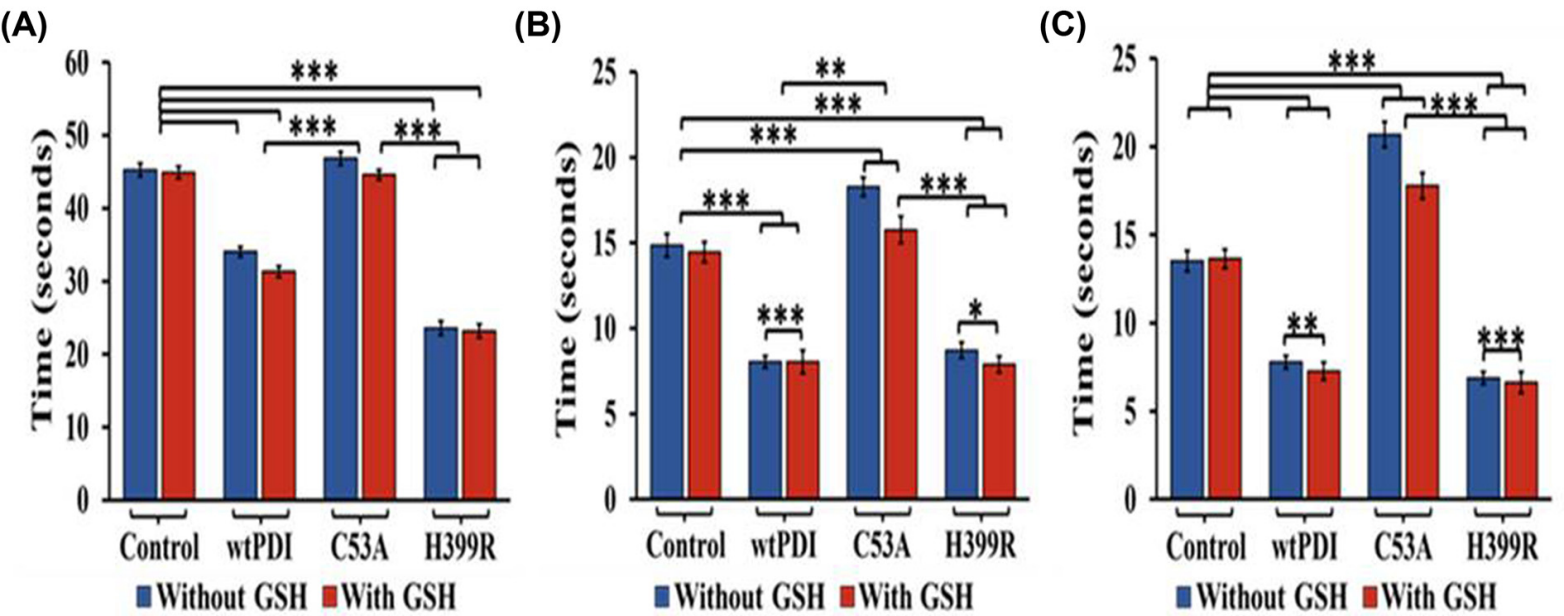
Effect of exogenous PDI and its variants on clotting time In the control set from A to C, the blue bar represents the plasma alone in the absence of reduced glutathione (GSH), and the red bar indicates the presence of GSH. While the set of wtPDI, C53A, and H399R having PDI, C53A, and H399R variant with plasma in the presence and absence of GSH, represented with red and blue color, respectively. (**A**) Activated partial thromboplastin time (APTT), (**B**) prothrombin time (PT), and (**C**) thrombin time (TT), of wtPDI, C53A, and H399R (175 nM). *P*-values of <0.05 were considered as significant with *P<0.05, **P<0.01 and ***P<0.001, ANOVA. Data are representative of at least five independent experiments (*n* = 5 in each group).

PDI with enhanced (H399R) and reduced (C53A) activity were used to check their influence on the blood coagulation rates and the results are summarized in Figure 4. It was found that the presence of GSH resulted in maintaining the PDI in its more reduced form. It helps the enzyme to make non-native disulfide bonds with their targets. However, we did not find any pronounced effect of GSH on wtPDI and its variant in clotting time assays. The results indicate that the reduced activity variant of PDI, namely C53A, did not significantly impact clotting time as compared with their native counterpart. The C53A mutant shows a delay in clotting by 3.49% in APTT, 22.49% in PT, and 53.14% in TT assay. However, The H399R variant demonstrated a notable reduction in clotting time in APTT, PT, and TT assays compared with the control, with percentage decreases of 47.96%, 41.38%, and 49.7%, respectively (Figure 4 and Supplementary Table S4). This shows that the decrease in the clotting time was more significant in the TT (Figure 4C), but APTT (Figure 4A) and PT (Figure 4B) were also affected. In all cases, increased activity of PDI results in a greater influence on the rate of coagulation.

### Clotting times in the presence and absence of activator and inhibitor

**Figure 5:**
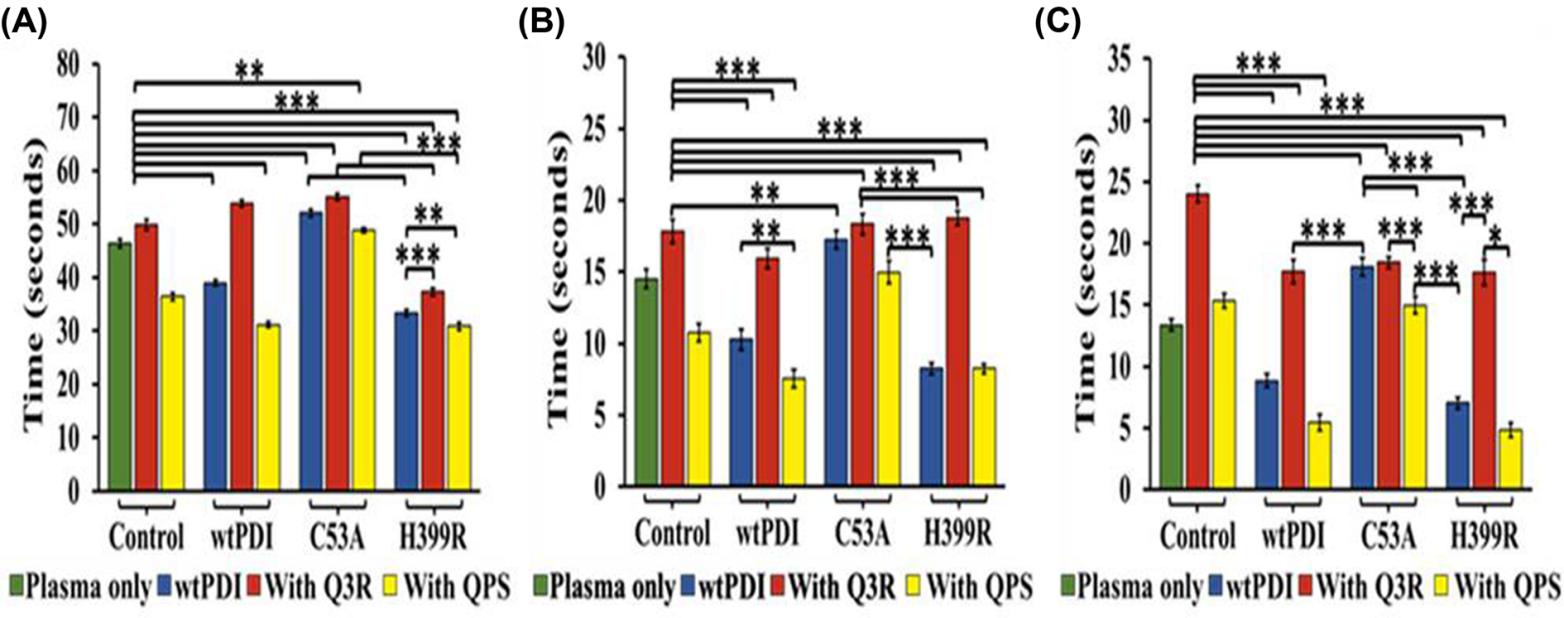
Effect of exogenous PDI and its variants on clotting time with Q3R and QPS In the control set, from A to C, the green bar represents the negative control with platelet-poor plasma (PPP) only, while the red and yellow bars represent PPP with Q3R and QPS, respectively. Within the wtPDI, C53A, and H399R set, the blue bar represents wtPDI, C53A, and H399R in platelet-poor plasma. The red bar denotes the presence of Q3R with PPP and wtPDI, C53A, and H399R, and the yellow bar indicates the incubation of QPS with PPP and wtPDI, C53A, and H399R. (**A**) Activated partial thromboplastin time (APTT), (**B**) prothrombin time (PT), and (**C**) thrombin time (TT) show a marginal delay in clotting time in C53A and H399R (175 nM) mutants in the presence of Q3R (100 µM). Additionally, QPS shows a marginal alteration in clotting rate upon incubation with C53A, whereas incubation of H399R with QPS (100 µM) decreases the clotting time. *P*-values of <0.05 were considered as significant with *P<0.05, **P<0.01 and ***P<0.001, ANOVA. Data are representative of at least five independent experiments (*n* = 5 in each group).

The addition of activator QPS in the presence of H399R decreased the blood clotting time in APTT by 15.25%, 23.42% in PT assay, and 68.40% in the TT assay, and the addition of Q3R also delayed the time of clotting (Supplementary Table S5). The results confirm the direct modulation of the blood coagulation rates by exogenous addition of the PDI variants (Figure 5A–C). The results also indicate that the thrombin time is more affected as compared with the APTT and the PT times and pointed to the important role of the thrombin in the PDI-based enhancement in the clotting time.

### PDI binds to the thrombin–antithrombin complex and retains its activity

**Figure 6:**
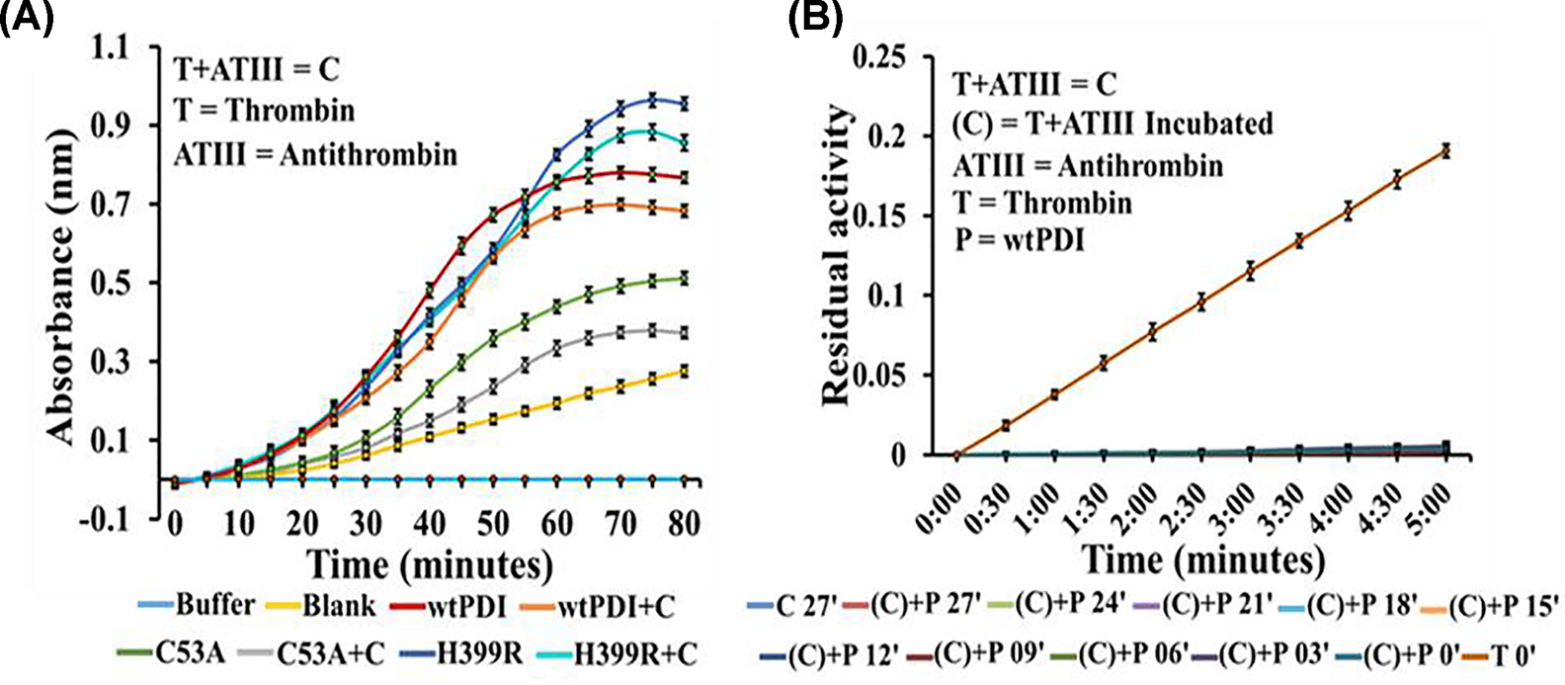
Antithrombin–thrombin complex and PDI specificity Panel (**A**) represents the effect of T-ATIII preformed complex on wtPDI, C53A, and H399R insulin reductase activity. Panel (**B**) shows the specificity of wtPDI in antithrombin–thrombin inhibition assay, in which thrombin and antithrombin were incubated for 15 min and wtPDI was added in different time intervals from 0 to 27 min, as control thrombin alone was also plotted in the figure that represents the classical antithrombin associated thrombin inhibition assay in presence of thrombin specific chromogenic substrate S-2238 at different time intervals.

Insulin reductase assay was conducted under reducing conditions with PDI and its variants in the presence of ATIII–thrombin complex. Based on the results, it can be observed that the wtPDI, as well as the H399R and C53A variants, exhibited a slight decrease in their activity when the ATIII–thrombin complex was present (Figure 6A). Under similar conditions, thrombin was completely inhibited by the ATIII (Figure 6B). PDI was incubated with the ATIII–thrombin complex, as well as thrombin and ATIII individually. A Western blot analysis revealed that complex formation occurred only with the ATIII–thrombin combination. Consequently, PDI incubation with thrombin or ATIII didn’t affect the thrombin inhibition rates (data not shown). The results indicate that PDI and ATIII activity is maintained on binding the ATIII–thrombin complex.

### PDI uses a thrombin–antithrombin complex to influence the plasma substrate

**Figure 7:**
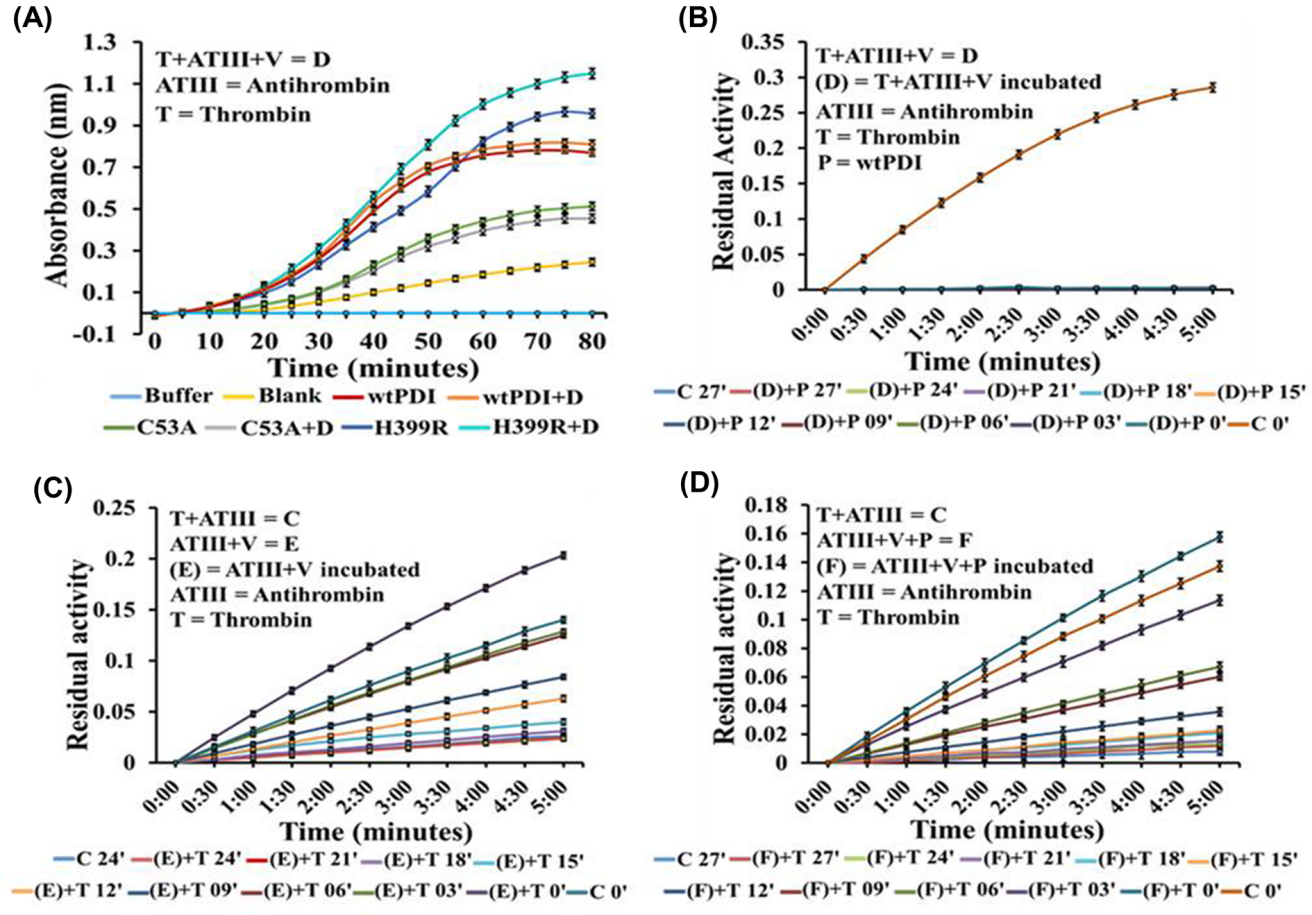
Activity analysis of ATIII–thrombin–PDI complex (**A**) Represents the impact of thrombin–ATIII–vitronectin complex on wt-PDI, C53A, and H399R insulin reductase activity. Panel (**B**) represents the impact of the thrombin–ATIII–vitronectin complex on antithrombin inhibition of thrombin. Panel (**C**) represents the ATIII inhibition of thrombin in the presence of vitronectin without the ATIII–thrombin template formation; panel (**D**) is the inhibition of thrombin inhibition by antithrombin in the ATIII–thrombin–vitronectin–PDI complex. The results indicate no compromise in antithrombin activity in complex with thrombin, and also in the presence of PDI and vitronectin. Each experiment is S.E. of at least three independent experiments.

Vitronectin is a PDI substrate detected using the PDI trap variants that influence thrombus formation. We examined how the binding of PDI and vitronectin to the ATIII–thrombin complex influences the activities of PDI and thrombin, and the results are summarized in Figure 7. Insulin turbidity assay of PDI, H399R, and C53A variant on binding to the ATIII–thrombin complex in the presence of vitronectin showed that PDI was fully active (Figure 7A). Under the same condition ATIII was still able to completely inhibit the thrombin (Figure 7B). The ATIII on incubation with vitronectin (Figure 7C) or PDI (Figure 7D) can effectively inhibit thrombin indicating that the anticoagulant role of the ATIII is maintained in isolation as well as on complex with the ATIII–thrombin–PDI.

### Western blot analysis of antithrombin–thrombin–PDI complex

**Figure 8:**
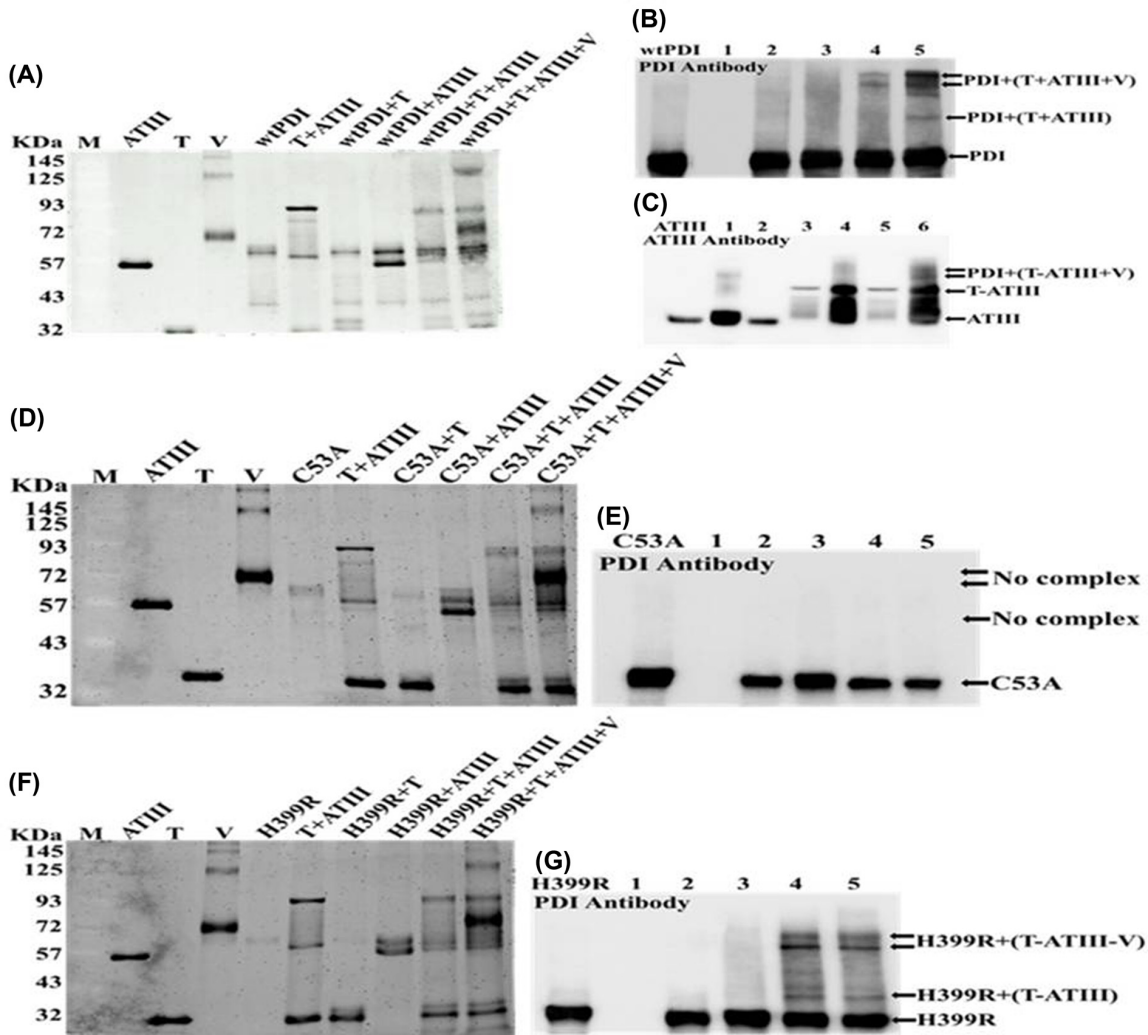
Assessment of ATIII–FIIa–wtPDI complex Panel (**A, D, F**) represent the complex formation between PDI and its variants with thrombin, antithrombin, and vitronectin. Lane M is a protein marker of 11–180 kDa. ATIII-FIIa complexes were formed in the presence of heparin and wtPDI, C53A, and H399R, with thrombin and antithrombin in the redox environment provided with GSH. Panels (**B, E, G**) show the Western blot with monoclonal anti-PDI antibody, where **Lane 1** is ATIII-FIIa complex (not detected with anti-PDI antibody), **Lane 2** of B, E, and G is wild-type PDI, C53A, and H399R with thrombin, in **Lane 3** native PDI, H399R and C53A is with antithrombin, respectively. wtPDI, C53A, and H399R were incubated with preformed ATIII-FIIa complex in **Lane 4** of B, E, and G, **Lane 5**, shows the V-ATIII-FIIa complex in the presence of wtPDI, C53A, and H399R in B, E, and G, respectively. Panel (**C**) represents Western blot using a monoclonal anti-ATIII antibody. Antithrombin with wtPDI, vitronectin, and complexed with thrombin were represented from **Lanes 1–3**, respectively; in **Lane 4**, wtPDI was incubated with preformed ATIII-FIIa complex; **Lane 5** represents the thrombin and antithrombin with vitronectin. **Lane 6** involves the antithrombin, thrombin, and vitronectin together with reduced wtPDI.

The complexes with PDI and its variant were analyzed on SDS PAGE and were visualized using ATIII and PDI antibodies. PDI doesn’t interact with ATIII or thrombin, but it is able to bind the ATIII–thrombin template and form a ternary complex (Figure 8A). PDI and ATIII antibodies were used to show that PDI and ATIII are part of the ternary as well as quaternary complexes (Figure 8B,C). Under similar conditions, C53A variant didn’t show any complex formation in the presence or absence of vitronectin (Figure 8D,E). However, H399R is also able to form a complex with ATIII–thrombin similar to that observed with wt PDI (Figure 8F) and confirmed by the Western blot analysis (Figure 8G). The results establish that the PDI and ATIII are fully active in the ATIII–thrombin–PDI complex and can bind to the plasma substrate.

## Discussion

The modulation of thrombin by PDI presents an alternative therapeutic possibility for regulating thrombosis and its associated pathological phenotypes. Direct and indirect inhibitors of blood coagulation are burdened with a multitude of drawbacks, primarily stemming from the cross specificity of heparin-based drugs and the variability in the dose response [22,24]. Understanding the mechanism of interaction of the plasma substrates with PDI, under the conditions in which it catalyzes oxidation, reduction, or isomerization, as well as the resulting changes in targets or newly acquired functions, holds significant importance [25]. Therefore, it is critical to decipher the mechanisms and pathways that influence the coagulation cascade indirectly and have the potential to modulate thrombosis. PDI is predominantly prothrombotic and ATIII is a central component of anticoagulation control through its inhibition of the (factor Xa) FXa, thrombin, (factor XIa) FXIa, and (factor IXa) FIXa [26,27]. Therefore, the influence of PDI on ATIII may provide clues to its regulation during inflammatory, stress, or hypoxic conditions, and the situations that are conducive to its release in plasma [28].

In this study, we have tried to understand the molecular basis of PDI regulation of ATIII, a major anticoagulant protein of the serine protease inhibitor family (serpins). In a prior study, we presented the development of a derivative of quercetin (QPS) that exhibited a remarkable ability to substantially enhance the activity of PDI [29]. To determine the direct impact of PDI on the coagulation cascade, we conducted coagulation assays (APTT, PT, and TT) using its activator (QPS) and inhibitor (Q3R). The results demonstrated the ability to modulate the prothrombotic role of PDI (Figure 1). The H399R variant of PDI exhibited higher activity, leading to a decrease in the blood coagulation time. Conversely, the C53A variant increases the time of coagulation (Figures 4 and 5). APTT and PT were affected in both cases, suggesting modulation of both the intrinsic and extrinsic pathways. However, the most significant impact was observed in the TT, indicating the involvement of thrombin in the PDI modulation.

We show that PDI binds to the ATIII–thrombin to form a stable ternary complex and is fully active (Figure 6A). This ternary complex also has full ATIII activity, and the thrombin activity is completely suppressed (Figure 6B). The ATIII–thrombin complex remains stable and can independently bind both PDI and vitronectin. Interestingly, this ternary complex also maintains the activity of both PDI (Figure 7A) and ATIII (Figure 6B), despite their seemingly contrasting role in coagulation (Figures 6 and 7). Using PDI and ATIII antibodies the formation of the ATIII–thrombin complexes was deciphered, C53A variant was unable to form the complex even in the presence of the ATIII–thrombin template (Figure 8). The ATIII–thrombin–PDI complex may catalyze the vitronectin disulfide exchange that has been shown to increase the interaction of vitronectin and integrins on the surface of endothelial cell and platelets to influence thrombosis [15,16]. Platelet surface-associated PDI regulates the binding of coagulation factors to platelets, a crucial process for generating thrombin [30]. The addition of exogenous PDI instantly makes the environment prothrombotic, therefore the levels of PDI bound to the ATIII–thrombin complex may critically determine the activation of the platelets, and the fully active complex bound ATIII will control the thrombin to maintain the hemostatic balance. It is also important to know if the ATIII–thrombin–PDI bound ternary complex can effectively bind to the platelet and induce coagulation factor binding and thrombin generation. Thus, based on the results of the above analysis following hypothesis is proposed.

### Possible mechanism of the PDI control of thrombosis

**Figure 9:**
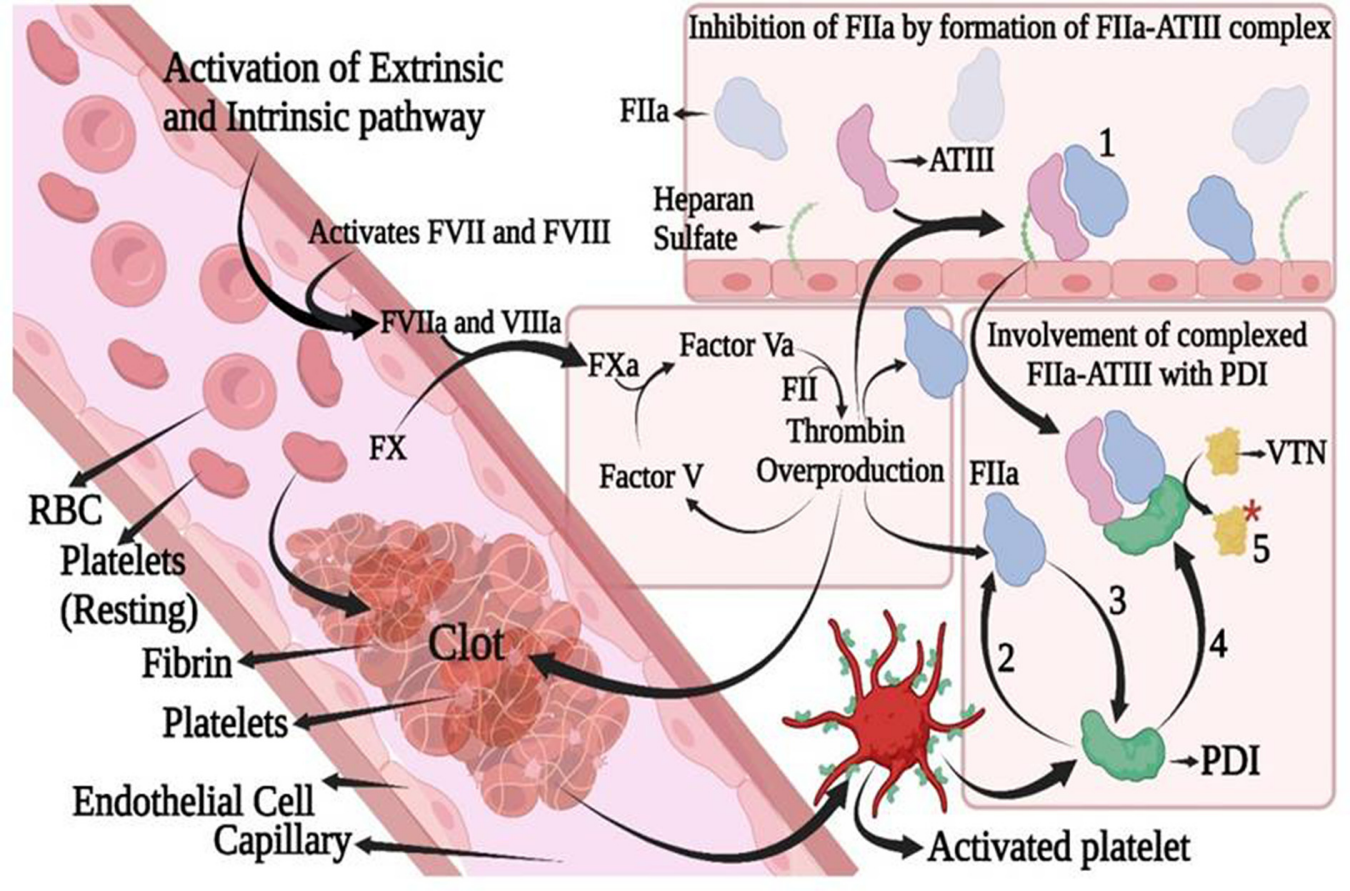
Mechanistic pathway showing involvement of PDI with thrombin (FIIa) and ATIII The image depicts the activation of coagulating factors via intrinsic and extrinsic pathways which in turn causes the blood to clot by following the series of activation steps. Overproduced FIIa will be removed from the system by its direct inactivation through the formation of the FIIa-ATIII complex in the presence of heparan sulfate (shown by numeral 1). Numeral 2 and 3 indicate that there is no effect of PDI on FIIa activity (no interaction observed). Numeral 4, shows that PDI will interact with the FIIa-ATIII complex and retains its full activity and ATIII is also fully active. PDI ternary complex with FIIa–ATIII will introduce disulfides within the vitronectin (represented by the numeral 5), to enhance thrombosis.

The intrinsic and extrinsic coagulation control pathways merge in the critical formation of thrombin from prothrombin using factor Xa (Figure 9). Presence or absence of thrombin marks the prothrombotic or anticoagulant environment of the plasma. ATIII is the major inhibitory heparin-dependent serpin that controls thrombin levels. In the presence of heparin or other sulfated molecules, thrombin levels are controlled by the formation of the ATIII–thrombin complex (numeral 1). PDI activity is not affected in the presence of thrombin (numerals 2 and 3), and the environment is largely prothrombotic. Free thrombin starts to form complexes with ATIII and is completely inhibited, ATIII is completely active and PDI also retains its activity on binding to the ATIII–thrombin complex (numeral 4). PDI is also fully active on binding to the ATIII-thrombin template in the presence of its substrate target like vitronectin and forms a quaternary complex and may catalyse the disulfide switch (numeral 5), the plasma substrate on the disulfide switch influences thrombosis. Heparin cofactor II, protein C inhibitor, and plasminogen activator inhibitors are also the serpins involved in coagulation control that have thrombin as their proteolytic target. Therefore, it is hypothesized that the serpin–thrombin complexes might be the common templates that PDI uses to perform disulfide switches to influence thrombosis. However, further elaborative studies are needed to test the role of serpin-thrombin complexes and their modulation by PDI to influence thrombosis.

## Supplementary Material

Supplementary Figures S1-S3 and Tables S1-S5

## Data Availability

The data sharing is not applicable as all the supporting data are included within the main article and its supplementary files.

## Abbreviations

APTT: activated partial thromboplastin time
ATIII: antithrombin
ATIII-FIIa: antithrombin–thrombin
DTT: dithiothreitol
ER: endoplasmic reticulum
FIX: factor IX
FXI: factor XI
FV: factor V
GSH: glutathione
PDI: protein disulfide isomerase
PPP: platelet-poor plasma
PT: prothrombin time
TSP1: thrombospodin1
TT: thrombin time
