# Supplementary material for: Protein disulfide isomerase uses thrombin–antithrombin complex as a template to bind its target protein and alter the blood coagulation rates
Source: Biosci Rep. 2024 May 15;44(5):BSR20231540. doi: 10.1042/BSR20231540 (PMC11096647; doi:10.1042/BSR20231540)
Supplement: Supplementary Figures S1-S3 and Tables S1-S5 [file BSR-2023-1540_supp.pdf]

Thrombin-antithrombin complex based switch in protein disulfide isomerase and thrombin activity controls the coagulation rates: Implications in targeting thrombosis

Abdul Burhan Khan[1], Urfi Siddiqui[1], Sana Fatima[1], Ahmed Abdur Rehman[1] and Mohamad Aman Jairajpuri*[1]

[1]*Department of Biosciences, Jamia Millia Islamia, Jamia Nagar, New Delhi, 110025, India*

Supplementary Table 1: Primer sequence used for Site-directed mutagenesis in WT PDI.

Domain	Mutation	Mutagenic Primers	Oligonucleotide Sequence 5'-3'	Annealing Temperature
**a-domain**	**C53A**	TG163GC -FP	TGC GCC GTG G**GC** CGG TCA TTG	**69.0°C**
		TG163GC -RP	CAA TGA CCG **GC**C CAC GGC GCA	**69.0°C**
**a'-domain**	**H399R**	A1202G-FP	TGG TGC GGT C**G**C TGC AAG CAG	**67.3°C**
		A1202G-RP	CTG CTT GCA G**C**G ACC GCA CCA	**67.3°C**

Supplementary Table 2: Thermocycling conditions for PCR.

PCR REAGENT		PCR REACTION CONDITIONS			
**Reagent**	**Final conc.**	**Cycle Element**	**Temp (°C)**	**Time (sec)**	**cycles**
**5X High fidelity Reaction Buffer**	1X	Initial denaturation	94	300	1
**10 mM dNTPs**	100µM	Denaturation	94	40	16-18
**10µM Forward primer**<br>**10µM reverse primer**	0.1µM to 0.2µM	Annealing	Variable	40	
**Template DNA**	<100 ng	Elongation	72	480	
**Phusion high fidelity polymerase (2 U/µL)**	0.02 U/µL	Final elongation	72	600	1
**Distilled Water**	Up to 100µL	Hold cycle	4		

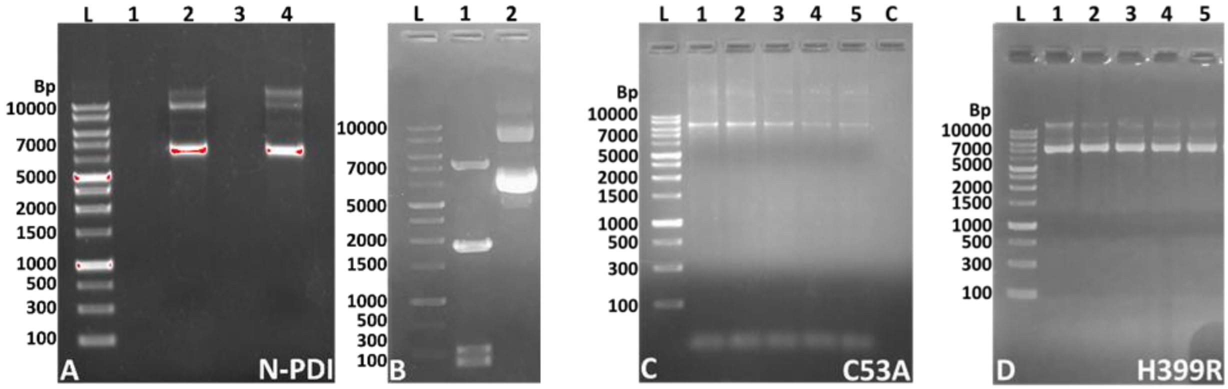

Supplementary Figure 1: Agarose gel electrophoresis. (A) Agarose gel electrophoresis of isolated PDI gene (Lane-L represents DNA ladder, Lane-2, and 4 are isolated PDI DNA plasmid, Lane 1 and 3 (left blank). **(B)** Agarose gel 0.8% (w/v) shows fall out of the PDI (Lane-L represents Ladder, Lane-1 Nhe I and Hind III digested product containing ~1539 bp gene, Lane-2 contains the intact inserted protein with vector) at ~6905 bp corresponding to PDI gene confirming the cloning of PDI gene in pET28b (+) vector. **(C and D)**, show the amplified products of all the designed mutants at different temperature gradients using native PDI as a template.

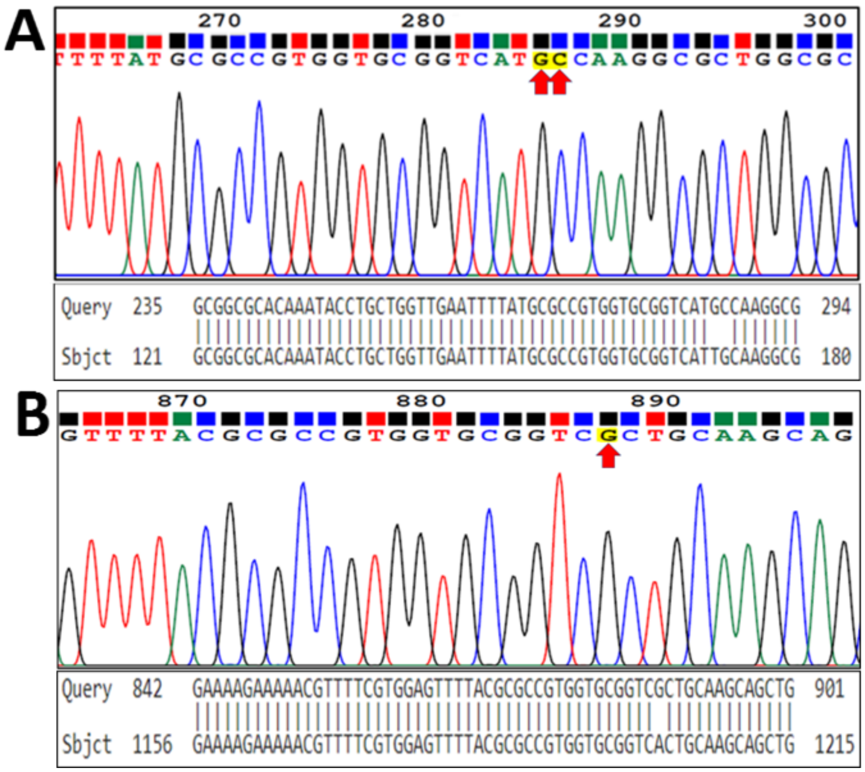

Supplementary Figure 2: Mutation confirmation using Sanger's sequencing. (A) reperesents the C53A mutation and (B) shows H399R mutation. Every red arrow in the chromatogram indicates the position of successfully substituted bases in place of the template sequence. Below every chromatogram is the BLAST results, which are aligned against the template sequence denoted as "subject" and the amplified product sequence was mentioned as "query".

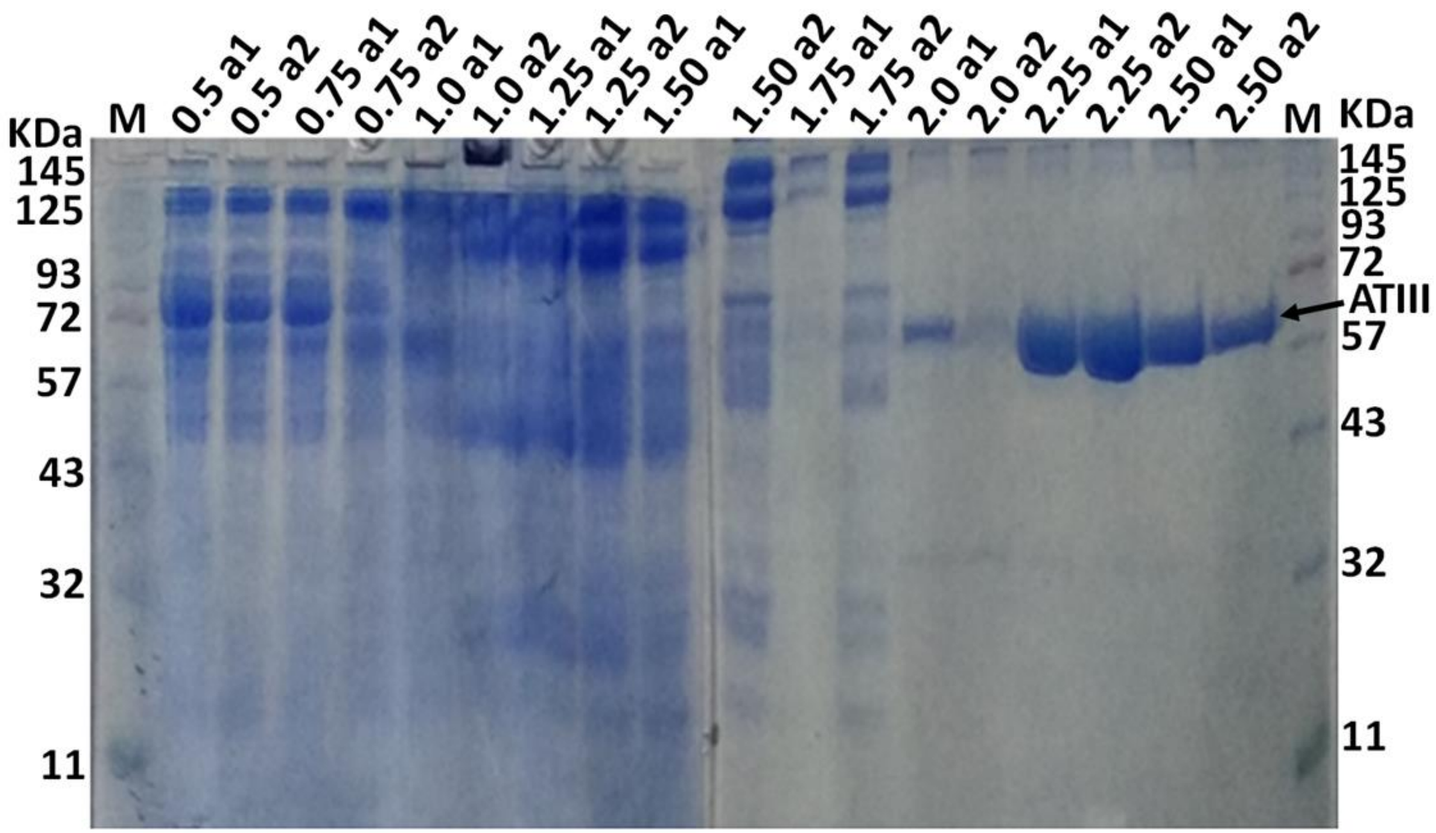

Supplementary Figure 3: Purification of Antithrombin from Human plasma. The protein fractions were collected in a set of 2 with same concentration labelled as "a1" and "a2". Protein was eluted from 0-3M of NaCl gradient. Monomeric ATIII was obtained in fraction 2.25a1-2.50a2. Lane M indicates the protein marker ranges from 11-180KDa.

Supplementary Table 3: APTT, PT, and TT coagulation assays clotting time in seconds (for Fig. 1)

		Plasma only	Protein only	With Q3R	With QPS
**APTT**	**Control**	46.823±1.071		49.373±1.08	36.618±1.084
	**wtPDI**		38.658±0.833	53.67±0.945	30.783±0.891
**PT**	**Control**	14.511±0.672		17.943±0.733	10.65±0.552
	**wtPDI**		10.238±0.792	15.936±0.704	7.576±0.616
**TT**	**Control**	13.376±0.453		23.96±0.784	15.31±0.598
	**wtPDI**		8.946±0.555	17.44±0.946	5.673±0.676

Supplementary Table 4: APTT, PT, and TT coagulation assays clotting time in seconds (for Fig. 4)

		Control	wtPDI	C53A	H399R
**APTT**	**Without GSH**	45.26±0.87	34.06±0.60	46.85±0.93	23.56±0.95
	**With GSH**	44.92±0.83	31.33±0.75	44.60±0.68	23.17±0.92
**PT**	**Without GSH**	14.86±0.67	8.05±0.34	18.28±0.55	8.72±0.47
	**With GSH**	14.45±0.59	8.05±0.67	15.77±0.79	7.89±0.47
**TT**	**Without GSH**	13.52±0.58	7.80±0.36	20.69±0.72	6.88±0.34
	**With GSH**	13.63±0.53	7.27±0.51	17.79±0.73	6.64±0.60

Supplementary Table 5: APTT, PT, and TT coagulation assays clotting time in seconds (for Fig. 5)

		Plasma only	Protein only	With Q3R	With QPS
**APTT**	**Control**	46.39±0.74		49.83±0.96	36.45±0.67
	**wtPDI**		38.99±0.55	53.83±0.66	31.18±0.59
	**C53A**		52.05±0.64	55.05±0.62	48.80±0.42
	**H399R**		33.33±0.59	37.31±0.67	30.89±0.72
**PT**	**Control**	14.51±0.67		17.83±0.83	10.76±0.62
	**wtPDI**		10.27±0.73	15.94±0.70	7.58±0.62
	**C53A**		17.26±0.62	18.33±0.71	14.98±0.80
	**H399R**		8.24±0.42	18.75±0.49	8.25±0.34
**TT**	**Control**	13.35±0.46		24.01±0.69	15.33±0.59
	**wtPDI**		8.85±0.56	17.7±0.95	5.46±0.68
	**C53A**		18.08±0.75	18.46±0.47	14.99±0.69
	**H399R**		7.03±0.47	17.63±0.99	4.85±0.56
